# Notes

**Published:** 1896-07

**Authors:** 


### Notes.

### SPECIAL REGULATIONS FOR THE SECOND PAN-AMERICAN MEDICAL CONGRESS, TO MEET IN THE CITY OF MEXICO ON THE 16TH, 17TH, 18TH AND 19TH OF NOV. 1896.

### ENROLLMENT.

ARTICLE 1. In order to be properly enrolled, each member of the Congress will pay to the Treasurer thereof in the City of Mexico, the sum of five dollars gold.

### GENERAL SESSIONS.

ART. 2. There will be one opening session, one closing and one intermediate session of a purely scientific character.

ART. 3. The opening session, which will be of a solemn character and presided over by the Supreme authority of the Nation, besides being attended by the members of the Congress, will also be attended by the members of scientific societies, and other distinguished persons who may be invited. The session will be opened with the report of the General Secretary.

This will be followed by a speech of welcome, pronounced by the President of the Congress.

Two members will then speak on scientific subjects, and they will be followed by a speech from the President of the Republic. It is strongly recommended that the scientific speeches should be of short duration.

The intervals between the speeches will be filled up with musical performances.

ART. 4. At the closing session, the General Secretary will notify the place designated by the Congress for holding the third meeting.

ART. 5. The Treasurer will present his accounts to the Congress, showing the disbursements made of the funds entrusted to his care.

ART. 6. A scientific speech will be delivered and a short speech by one representative of each one of the nations attending the Congress.

ART. 7. In the intermediate session, four speeches will be delivered on general matters, by persons who are highly distinguished in medical science, and who, having been in due time invited to do so, have accepted the commisson; one of these speeches being pronounced by a Mexican physician, who shall be invited to do so by the Committee of Management.

ART. 8. No discussions will be held in the General Sessions.

SESSIONS OF THE SECTIONS.

ART. 9. These sessions will be held from 9 to 12 A. M. and from 3 to 5 P. M., in the places that may be designated by the Organizing Committee. They shall be presided over by the President of each section, alternating with the Vice-Presidents of each one of the nations that are represented in the respective sections.

ART. 10. The person who may be appointed by the Committee of Organization, will be the *ex-officio* Secretary of each section, and he will fill his post alternately with the Secretaries of the nations who may be represented in the sections; but should the latter not be present, their places will be supplied by the President in office.

ART. 11. The President will direct the discussion in accordance with the order of the day, and will decide all questions that may arise, and that may not be provided for in these regulations.

ART. 12. The *ex-officio* Secretary will make out the minutes and for that purpose, besides his own notes, will collect those of the Secretaries who may have acted in the sections· He will also collect from the persons who may have spoken, the written extracts referred to in ART. 16.

ART. 13. All questions relating to the debates, which are not provided for in these Regulations, will be decided in accordance with general parliamentary practice.,

ART. 14. The voting will be by name or by putting the question.

PAPPERS, EXTRACTS THEREOF AND DISCUSSIONS IN THE SESSIONS OF THE SECTIONS.

ART. 15. All papers will be presented in writing.

ART. 16. Each author will forward to the Secretary of the Organizing Committee in the City of Mexico, and before the first day of August of the present year, an extract not exceeding 300 words, of the paper to be presented by him. These extracts will be printed in English, French and Spanish, and will be distributed to the members of the Congress before the session in which they are to be read.

ART. 17. No paper will be announced which is not accompanied by this extract; but the authors who comply with these conditions will have a right to have their work published intact in the transactions of the Congress.

ART. 18. The reading of the papers in the sessions must not last more than twenty minutes; when the papers are so long that they cannot be read within that time, the authors will give extracts from them, either in writing or by speech; but they will be published intact in the transactions of the Congress, and in the language in which they have been written.

---

### VIVISECTION.

### FROM THE MINUTES OF THE AMERICAN ACADEMY OF MEDICINE FOR MAY, 1896.

(Advance sheet from the Bulletin of the Academy.)

The following resolutions, presented by Dr. Gould, and recommended by the Council, were discussed and adopted unanimously:

*Resolved*, That the American Academy of Medicine desires to express its opinion that no legislation is required or desirable in the United States in regard to the so-called practice or subject of "Vivisection," and for the following reasons:

1. Because only by careful discrimination and collection of facts can public and legislative opinion be truthfully formed. To illustrate but a single of many popular errors upon this subject: the dissection or use of dead aniamls by scientific men is termed "vivisection," when the same proceeding carried out by the butcher, the hunter, the restaurant keeper, cooks, etc., does not enter into the consideration, neither do the cruelties in the use and keeping of domestic animals, nor those in the deaths of animals for other purposes than those of experimental medicine

2. While admitting and deploring the facts of abuses in the past and in some European countries,—to a very limited extent also in America—it is the conscientious belief of the members of the Academy that at present with us such abuses do not exist, nor are they in danger of occurring, to a degree justifying or calling for legislation as, under the circumstances, the evils that would inevitably result from such legislation would greatly exceed the benefits to be obtained by it. The charge implied or openly made that physicians, either in theory or in practice are more cruel than other classes of the community, is a fancy or prejudice of ignorance which cannot be proved, and which we strenuously deny.

3. Legislation upon the subject of cruelty to animals should be so framed as to include consideration of cruelties infinitely greater and more extensive in many other fields of human activity at present not actively objected to by those who urge legislation as regards experimental medicine. Not only this, but legislation concerning these matters should be broadened out in order to prevent the destruction of species of birds and other animals by the votaries of fashion, by the hunters, etc., to prevent derangement of the delicate balance of animal and vegetable life upon which civilization ultimately and largely rests, to prevent deforestation of the head-waters of our streams, to establish sanctuaries or resorts for animals, and many such biologic requisites,—as also to establish such arrangements with other nations as will ensure their permanent and extensive effectualization.

4. Legislation upon a subject of vital importance to a peculiarly technical branch of sciences should be framed under the guidance and by the aid of those who by education and experience are alone fitted and capable of forming and expressing sound judgment upon it, *i. e.*, the experts in the special subject. It would be as absurd to have legislation as to vaccination, inspired and shaped by laymen who were anti-vaccinationists, without weighing the opinion of the medical profession, as to allow legislation upon the question of vivisection by laymen who are anti-vivisectionists, and even inexpert in any branch of inductive science.

5. The American Academy of Medicine therefore urges its members and physicians generally to write to their representatives in Congress, (or wherever legislation of the kind in question is proposed), and otherwise seek to influence public and official opinion against the passage of a particularly ill-advised bill before Congress, to wit, Senate Bill No. 1552, introduced by Mr. McMillan, entitled "A Bill for the Further Prevention of Cruelty to Animals in the District of Columbia." In the opinion of the Academy the pasage of this bill would be harmful to the true interests of medical and social science and to the public health.

---

The Mississippi Valley Medical Association.—A meeting of the Executive Committee of the Misissippi Valley Medical Association was held at Atlanta, on May 6th, and the following gentlemen were appointed to deliver addresses: Dr. H. N. Moyer, Chicago, Address on Medicine; Dr. Horace H. Grant, Louisville, Address on Surgery. The indications are that the meeting to be held at St. Paul, on October 20, 21, 22 and 23, will be the largest and most successful in the history of the Association. As all the railroads will offer reduced rates for the round trip, an opportunity will be given to visit St. Paul and Minnesota during the most delightful season of the year.

C. A. WHEATON, M. D., St. Paul, Minn.,
Chairman Committee of Arrangements.
H. O. WALKER, M. D., Detroit, Mich., President,
H. W. LOEB, M. D., 3559 Olive Street, St. Louis, Secretary.

---

*The Texas Medical Journal*, or the "Red Back," as its editor affectionately styles it, is just now greatly exercised over insinuations cast upon it by a contemporary medical publication in the same State. Texas is a big State, but it does not include the earth, and there are a few people left to whom the affairs of Dr. Daniel and the *Texas Medical News* are of no consequence whatever. Why not hire a hall and talk it out. It would settle the matter quickly and would save the non-union printer of the "Red Back" a big lot of work. We notice that Dr. Daniel is a dermatoloigst; perhaps this accounts for his bitterness.

Dr. S. G. C. Pinckney has been appointed lecturer on diseases of the mind and nervous system at the Southern Medical College. The following changes have been made in the faculty of the Southern Medical College: Dr. W. S. Crowe elected Professor of Obstetrics and Clinical Gynecology; Dr. J. G. Garnest, Professor of Gynecology; Dr. J. C. Olmsted, Prof. of Physiology; Mr. E. L. McCandless, Prof. of Chemistry; Dr. C. D. Hurt, Prof. of Materia Medica; Dr. Floyd W. McRae, Prof. of Diseases of the Rectum.

---

Professor Dr. Don Francisco Bastillos, Calle de Tacuba No. 7, Ciudad de Mexico, D. F., Republaca Mexicana, has been elected Treasurer of the Second Pan-American Medical Congress to be held in the City of Mexico the 16th of November.

All members residing in the United States and Canada, and others, who contemplate attending, should forward the registration fee, $5, gold, to him at once, and notify Dr. C. A. L. Reed, Cincinnati.

---

Dr. H. F. Harris has resigned the chair of chemistry in the Southern Medical College to accept the adjunct professorship of pathology at the Jefferson Medical College, Philadelphia. It is with profound regret that we regard this contemplated change, for Atlanta will lose thereby her most skillful pathologist, and the cause of higher medical education in the South one of its most active and efficient supporters. We wish for Dr. Harris every success in his enlarged sphere of usefulness.

---

Dr. Hunter P. Cooper has been elected to the chair of anatomy and clinical surgery at the Atlanta Medical College, to fill the vacancy occasioned by the death of Dr. W. S. Armstrong. Dr. Cooper is a surgeon of ability and large experience, and will prove a worthy successor to the lamented Armstrong.

---

Dr. L. B. Grandy has gone to New York to take a course in the eye, ear, nose and throat.

